# Seroprevalence of Crimean-Congo Hemorrhagic Fever and Rift Valley Fever Viruses Among Ruminants in Nigeria: A Descriptive Epizootiological Analysis

**DOI:** 10.3390/pathogens14121219

**Published:** 2025-11-30

**Authors:** David Odion Ehizibolo, Olumuyiwa Oyekan, Nicodemus Mkpuma, Dorcas Amara Gado, Isa Zayyad Turaki, Habibu Haliru, Ibrahim Garba, Elizabeth Ene Williams, Samdi Kennedy, Ardo Abdullahi, Bala Akawu, Banenat Bajehson Dogonyaro, Joshua Mallum Shallangwa, Caleb Saul Kilyobas, Innocent Gregory, Nuhu Auta, Moses Hyellafiya Kussiy, Abdullahi Mohammed, Musa Abdullahi Muhammad, Mansur Abubakar, Aminu Shittu, Maryam Muhammad, Corrie Brown, Bonto Faburay

**Affiliations:** 1 National Veterinary Research Institute, Vom 930001, Plateau State, Nigeria; 2 Ministry of Livestock and Aquaculture Development, Yola 640230, Adamawa State, Nigeria; shallangwajoshua@gmail.com (J.M.S.);; 3 Veterinary Department, Ministry of Agriculture and Natural Resources, Dutse 720101, Jigawa State, Nigeria; 4 Department of Veterinary Services, Ministry of Animal Health and Fisheries, Sokoto 840103, Sokoto State, Nigeria; 5 Quantitative Epidemiology and Animal Health Group, Department of Theriogenology and Animal Production, Faculty of Veterinary Medicine, Usmanu Danfodiyo University, Sokoto 840103, Sokoto State, Nigeria; shittuaminu38@gmail.com; 6 LifeStock International, 550, Fortson, Rd., Athens, GA 30606, USA; corrie@lifestock.org; 7 Foreign Animal Disease Diagnostic Laboratory, National Veterinary Services Laboratories, National Bio and Agro-Defense Facility, United State Department of Agriculture, Manhattan, KS 66505, USA; bonto.faburay@usda.gov

**Keywords:** Crimean-Congo hemorrhagic fever, Rift Valley fever, livestock, seroprevalence, zoonoses, Nigeria, One Health

## Abstract

Crimean-Congo hemorrhagic fever (CCHF) and Rift Valley fever (RVF) are major zoonotic diseases, spread by arthropods, with livestock serving as amplifying hosts. Despite Nigeria’s large ruminant population and robust cross-border animal trade, data on the seroprevalence of the viral agents causing these diseases remain limited. A longitudinal serological survey was conducted in five major livestock markets across Nigeria. A total of 3450 animals (cattle, sheep, and goats) were tested for Crimean-Congo hemorrhagic fever virus (CCHFV) and Rift Valley fever virus (RVFV) antibodies using ELISA. Data on species, age, sex, animal origin, and tick infestation were collected and analyzed. Overall seroprevalence was 27.1% (95% CI: 25.6–28.6) for CCHFV and 5.8% (95% CI: 5.1–6.7) for RVFV. Cattle showed the highest prevalence for both CCHFV (55.4%) and RVFV (11.2%), followed by sheep (17.4% and 2.9%) and goats (8.6% and 3.4%). Evidence of mixed exposure to both CCHFV and RVFV antibodies was detected in 8.2% of cattle, 0.7% of sheep, and 0.2% of goats. Seropositivity was higher in older animals, females, tick-infested animals, and those of Nigerian origin compared to imported animals. Market-level variation was observed, with Mubi livestock market showing the highest CCHFV prevalence (35.5%) and Illela livestock market the highest RVF prevalence (11.2%). The detection of con-current CCHFV and RVFV antibodies, alongside high CCHFV prevalence and detectable RVFV circulation among Nigerian livestock highlight the risk of zoonotic spillover, particularly in livestock markets with intense human–animal interaction.

## 1. Introduction

Crimean–Congo hemorrhagic fever (CCHF) and Rift Valley fever (RVF) are important emerging and re-emerging zoonoses with significant implications for both public and animal health. CCHF is caused by an RNA virus from the *Orthonairovirus* genus within the *Nairoviridae* family and is primarily transmitted through bites of *Hyalomma* ticks or direct contact with blood or tissues of infected animals, as well as via human-human transmission through exposure to body fluids [1,2,3]. The disease in humans is characterized by a potentially fatal hemorrhagic syndrome, with case fatality rates ranging from 5% to 62% depending on the outbreak and health system response [4,5]. RVF, on the other hand, is caused by a Phlebovirus (family *Phenuiviridae*) transmitted mainly through mosquito vectors (*Aedes* and *Culex* spp.) and through contact with infected animal products [6,7]. In livestock, RVF causes high abortion rates and neonatal mortality, particularly in cattle, sheep, and goats, while humans present with febrile illness, and in severe cases hemorrhagic, ocular, or neurological complications [7,8,9,10]. Both diseases are recognized as priority zoonoses due to their epidemic potential, high socio-economic impact, and occupational risk to veterinarians, butchers, abattoir workers, and farmers. The detection of antibodies against both diseases in domestic ruminants holds significant epidemiological importance, as it indicates the extent of virus circulation within animal populations and highlights potential spillover risks to humans [11].

Nigeria has one of the largest livestock populations in sub-Saharan Africa, with ruminants forming a cornerstone of livelihoods, food security, and trade [12]. Nigeria’s ruminant population in 2022/2023 comprised approximately 138.95 million goats, 64.93 million sheep, and 54.81 million cattle [13]. A recent study indicated that about 90% of the cattle population and 70% of the sheep and goat populations in Nigeria are concentrated in the northern region [14]. Livestock marketing and transboundary animal movements are common, with large markets serving as aggregation hubs for cattle, sheep, and goats from within and outside the country [15]. Importantly, Nigeria is also a major destination for livestock across West Africa, with estimates suggesting that up to 60% of all livestock in the region eventually enter Nigeria to meet the demands of its large and growing population. This tremendous importation means that sampling animals at Nigeria’s international livestock markets not only reflects local disease dynamics but also provides a unique snapshot of infection status and pathogen circulation in neighboring countries. These dynamics create conditions conducive to the spread of transboundary zoonoses such as CCHF and RVF. Seroprevalence studies conducted in Nigeria have shown that CCHFV occurs at rates ranging from 20 to 71.9% in cattle [16,17,18,19,20,21], 2 to 10.8% in goats [16,19], and 7.6% in sheep [19]. Similarly, RVFV seroprevalence has been reported at 10.2 to 17.2% in cattle [22,23,24,25,26], 4.5 to 18.7% in sheep [22,26,27], and 6 to 10.4% in goats [22,23]. However, these studies were based on relatively small samples sizes, were less comprehensive, and were often conducted in limited geographic areas, which may limit generalizability of their findings. Evidence suggests that tick vectors for CCHFV are widespread across Nigeria, while climatic conditions and vector ecology favor the potential establishment of RVF outbreaks [16,21,28,29]. Furthermore, the porous nature of regional borders, coupled with limited regulation and oversight in many livestock markets, facilitates frequent, large-scale animal movements. While these dynamics sustain trade and food security, they also amplify the risk of introducing and spreading zoonotic pathogens across borders, underscoring the importance of surveillance in these market systems.

A descriptive epidemiological approach is therefore warranted to provide prevalence estimates and to identify groups and settings at highest risk. Such evidence is critical to guide targeted surveillance, inform veterinary and public health interventions, and lay the foundation for subsequent analytical modeling and One Health studies. This study therefore conducted a comprehensive epizootiological survey to characterize the prevalence and spatial patterns of CCHFV and RVFV exposure in ruminant populations within livestock markets spanning multiple ecological zones in Nigeria.

## 2. Materials and Methods

### 2.1. Study Design and Setting

**Figure 1:**
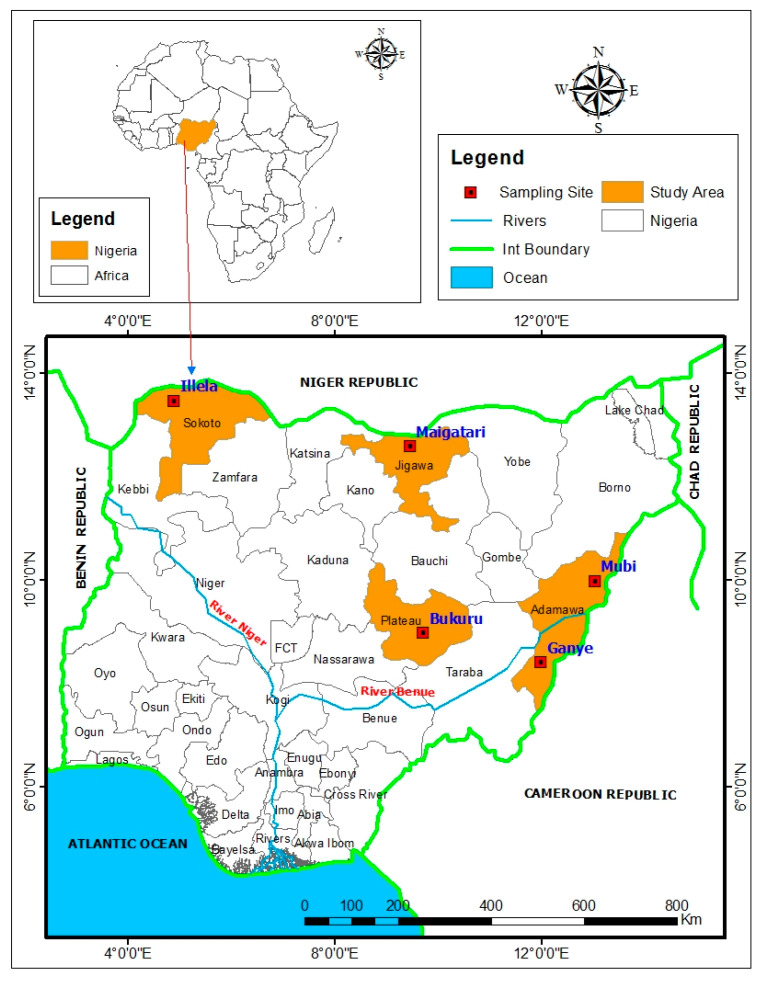
Map of Nigeria showing states of sample collection.

This project employed a longitudinal study design to determine the seroprevalence of CCHF and RVF among ruminant livestock through an active surveillance conducted in five major livestock markets situated in Nigeria’s North West, North East states, and a North Central state (Figure 1). These sites were purposively chosen because they represent key nodes for livestock trade and cross-border movement with neighboring countries, including Niger, Cameroon, and Chad. In the North West, sampling was conducted at Illela (Sokoto state) and Maigatari (Jigawa state) livestock markets, while in the North East, sampling occurred in Mubi and Ganye (Adamawa state) livestock markets. In North Central, the Bukuru livestock market (Plateau state) was the site for sampling.

### 2.2. Study Population and Data Collection

A total of 3450 ruminant animals were sampled for CCHFV and RVFV testing. Animals were stratified by species, age (young vs. adult), sex, origin (local vs. foreign), and tick infestation status. The study population consisted of three ruminant species commonly traded and reared in Nigeria: cattle, sheep, and goats. For each animal sampled, information was collected on demographic characteristics (species, age, sex), and trade-related attributes (market location, origin of animal).

### 2.3. Sample Collection

During weekly visits, for a period of 45–47 weeks during June 2023–July 2024, a total of 1150 animals from each species were sampled using a convenient sampling method. At each sampling location, at least five blood samples per species were collected aseptically from the jugular vein of each animal using sterile disposable needles and vacutainer tubes without anticoagulant. Approximately 5–10 mL of blood was drawn per animal. Samples were labeled with unique animal identification numbers and immediately stored in cool boxes containing ice packs. Samples were transported to the nearest field laboratory where they were allowed to clot, after which sera were separated by centrifugation at 3000 rpm for 10 min. The harvested sera were aliquoted into cryovials, stored at −20 °C, and subsequently transported under cold chain conditions to the National Veterinary Research Institute (NVRI), Vom, Plateau State, Nigeria, for laboratory analysis.

### 2.4. Serological Analysis

All sera were screened for antibodies against CCHFV and RVFV using a validated commercial enzyme linked-immunosorbent assays (ELISA) kits (Innovative Diagnostics, Grabels, France) The tests were carried out in accordance with the indications for the ID Screen^®^ CCHF Double Antigen Multi-species and ID Screen^®^ Rift Valley Fever Competition Multi-species and IgG capture diagnostic kits designed to detect antibodies from the nucleoproteins (NPs) of CCHF and RVF viruses in the serum or plasma of ruminants. Each assay run included positive and negative control sera supplied with the kits to validate test performance. All sera and controls were tested at dilutions of 1:1.6 for CCHF and 1:1 for RVF, respectively, in accordance with the manufacturer’s instructions.

Optical density (OD) values were read using a microplate reader at 450 nm, and sample-to-positive (S/P) ratios or competition percentage values were calculated as specified by the manufacturer. Interpretation of results was conducted according to the kits OD ratios and cutoff values, with samples classified as positive or negative. Borderline or doubtful results (where applicable) were retested, and if consistently ambiguous, were excluded from final analyses. Final results for each animal were recorded in the dataset as binary outcomes (1 = positive, 0 = negative) for both CCHF and RVF.

### 2.5. Variable Definition and Coding

The primary outcomes were defined as CCHF and RVF seropositivity, coded as binary variables with 1 representing a positive test and 0 representing a negative test. Predictor variables were carefully defined and recorded for consistency and analytical robustness. For analytical purposes, age, based on dentition, was categorized into dichotomous groups: young (≤24 months for cattle, ≤12 months for sheep and goats) and old (>24 months for cattle, >12 months for sheep and goats), reflecting species-specific thresholds for maturity and potential cumulative exposure to infection. Sex was recorded as male or female. Origin was defined as either local (born and reared in Nigeria) or foreign (imported from neighboring countries) and presence of ticks as yes/no.

### 2.6. Data Management and Analysis

All data were cleaned and cross-checked for completeness before analysis. Descriptive statistics were generated for categorical variables as frequencies and percentages. Seroprevalence estimates for CCHF and RVF were calculated overall and across predictor categories, with 95% confidence intervals (CIs) computed using binomial exact methods, to show the statistical range within which the true prevalence is expected to fall. Comparative prevalence of CCHF and RVF was presented to highlight differences across species, sex, age, location, origin, and tick infestation. All statistical analyses were conducted using Stata/IC version 15.0 [30].

## 3. Results

### 3.1. Characteristics of the Study Animals

A summary of the distribution of the study population across explanatory variables is shown in Table 1. Species were almost equally represented, with cattle (*n* = 1150; 33.3%), goats (*n* = 1150; 33.3%), and sheep (*n* = 1150; 33.3%). Sampling was evenly distributed across the five livestock markets, ranging from 19.6% at Maigatari to 20.4% at Mubi. The age distribution was skewed toward older animals (≥2 years for cattle, >12 months for sheep and goats), which made up 85.9% (*n* = 2963), compared with 14.1% (*n* = 485) for younger animals. Females predominated in the sample (64.0%, *n* = 2208) compared with males (34.7%, *n* = 1196), while 46 animals (1.3%) had missing (not recorded) sex information. Regarding origin, 69.9% were local animals, 29.3% were from neighboring countries, and 0.8% had missing data. Tick infestation was detected in 13.0% of animals, with 85.6% free of ticks. Overall, Table 1 highlights that the study population was broadly balanced across species and locations, though dominated by older and female animals.

### 3.2. Serological Prevalence of CCHFV in Ruminants at Nigerian Livestock Markets

A total of 3450 ruminant animals were screened for antibody against CCHFV (Table 2). The overall seroprevalence was 27.1% (95% CI: 25.6–28.6). Seropositivity was highest in cattle (55.4%, 95% CI: 52.5–58.1), moderate in sheep (17.4%, 95% CI: 15.3–19.7), and lowest in goats (8.6%, 95% CI: 7.1–10.3) (Table 2). Age-stratified analysis showed slightly higher prevalence in older animals (27.6%, 95% CI: 26.0–29.2) than in younger ones (25.5%, 95% CI: 21.8–29.6). Female animals demonstrated higher seropositivity (30.4%, 95% CI: 28.5–32.4) compared to males (21.6%, 95% CI: 19.4–24.1). With respect to animal origin, indigenous livestock had slightly higher seropositivity (29.3%, 95% CI: 26.6–32.2) compared to those imported from Niger, Chad, and Cameroon (26.2%, 95% CI: 24.5–28.0). Market-level variation was observed, with the Mubi livestock market recording the highest prevalence (35.5%, 95% CI: 32.0–39.1), while other markets showed relatively similar rates (Table 2). Animals carrying ticks had a higher seroprevalence of 40.1% (95% CI: 35.6–44.7) compared to 25.2% in tick-free animals.

### 3.3. Serological Prevalence of RVFV in Ruminants in Nigerian Livestock Markets

Of the 3450 ruminant animals tested across five livestock markets, the overall seroprevalence of Rift Valley fever (RVF) was 5.8% (95% CI: 5.1–6.7). Cattle exhibited the highest prevalence (11.2%, 95% CI: 9.5–13.2), followed by goats (3.4%, 95% CI: 2.5–4.6) and sheep (2.9%, 95% CI: 2.0–4.0) (Table 3). Seropositivity was nearly identical across age categories, with 6.2% (95% CI: 4.4–8.7) in younger animals and 5.8% (95% CI: 5.0–6.7) in older animals. Female animals showed a higher prevalence (6.7%, 95% CI: 5.7–7.8) compared with males (3.9%, 95% CI: 3.0–5.2). Animal origin and tick infestation status were also important predictors. Indigenous animals recorded a higher prevalence (10.6%, 95% CI: 8.8–12.6) than those imported from neighboring countries (3.8%, 95% CI: 3.1–4.6). Similarly, animals infested with ticks had a higher seropositivity (12.0%, 95% CI: 9.3–15.4) compared with tick-free animals (4.9%). Geographical differences were evident, with the Illela livestock market recording the highest prevalence (11.2%, 95% CI: 9.0–13.7), whereas Bukuru had the lowest (1.6%, 95% CI: 0.9–2.9).

### 3.4. Seroprevalence of Combined CCHFV and RVFV Antibodies in Ruminants in Nigerian Livestock Markets

The mixed-exposure analysis demonstrated the presence of antibodies to both CCHFV and RVFV across all ruminant species tested, although at varying levels. Overall, 104 of the 3450 (3.0%; 95% CI: 2.4–3.6) were seropositive for antibodies to both viruses. When examined by species, cattle exhibited the highest proportion of dual seropositivity, with 94 animals (8.2%; 95% CI: 6.6–9.8) testing positive to both CCHFV and RVFV antibodies (Table 4). In contrast, the occurrence of dual antibodies among sheep and goats was considerably lower. Only 8 sheep (0.7%; 95% CI: 0.22–1.18) and 2 goats (0.2%; 95% CI: 0–0.46) showed evidence of exposure to both viruses.

## 4. Discussion

This study provides sero-epizoological assessments of CCHFV and RVFV in ruminant livestock (cattle, sheep, and goats) across five major livestock markets in Nigeria. With four of these markets located at international borders, the findings provided serological evidence of these viruses circulating in a large West African region. The overall CCHFV seroprevalence of 27.3% observed in this study suggested sustained virus circulation among ruminant livestock in the region. This prevalence was consistent with findings from other, smaller studies in Nigeria and the region [16,19,31]. Our results showed that cattle had the highest CCHFV seropositivity, keeping in with reports from The Gambia, Cameroon, Senegal, Mauritania, Kenya, and Tanzania where cattle were consistently identified as having antibody to CCHFV [31,32,33,34,35]. The comparatively high seroprevalence may be explained by their larger body size, slower movement, greater body surface area and longer lifespan, which could increase their potential exposure to *Hyalomma* ticks, the primary vectors of CCHFV [34,36]. Sheep and goats showed markedly lower seroprevalence, which was in line with studies conducted in East Africa (Kenya and Tanzania) and North Africa (Tunisia), where small ruminants often showed lower seroprevalence compared to cattle [34,35,37,38]. The higher CCHFV seroprevalenece among animals from Mubi could be related to local ecological or management factors, such as higher tick density and livestock movement patterns. The overall seroprevalence of RVFV was relatively low, at 5.8%, which was consistent with findings from other West African countries, including Senegal (4.4%), and Cameroon (5.2%) [39,40,41], but markedly lower than levels documented in East Africa, where large-scale RVF outbreaks in Kenya, Tanzania, and Sudan have resulted in herd seroprevalence ranging between 15% and 40% [42,43]. The relatively low prevalence in Nigeria suggested limited virus circulation. Although a vaccine exists for RVFV, its use in West Africa is negligible, and so presence of antibodies was in all likelihood attributed to natural virus. Cattle similarly showed the highest RVFV seropositivity, comparable to previous report in Bauchi, Nigeria [24]. Goats and sheep had lower prevalence, similar to findings in Ghana, Niger, and Cameroon [44,45,46]. However, an earlier study in Nigeria reported slightly higher RVFV seroprevalence in sheep and goats compared to cattle [22]. Notably, animals with tick infestation showed comparatively higher seroprevalence compared to no-infested animals. This may reflect ecological overlap between mosquito abundance and tick presence, particularly in flood-prone grazing areas. There was geographic variability in RVFV antibody prevalence across markets, with highest detected in Illela and lowest in Bukuru. Illela, located in northwestern Nigeria, is ecologically closer to the Sahel and experiences seasonal flooding, which favors *Aedes* mosquito breeding. The overall pattern of the mixed exposure analysis indicated that concurrent or sequential exposures to both viruses are uncommon but present, particularly in cattle populations. Given that both CCHFV and RVFV are transmitted by arthropod vectors, ticks, and mosquitoes, respectively, the detection of animals with dual antibodies in livestock markets underscores the potential of co-endemic transmission zones where both vectors and pathogens overlap. The results suggested evidence of exposure to CCHFV and RVFV in Nigerian livestock and highlights the potential risk for zoonotic spillover, particularly in high-volume livestock markets that attract pastoralists and traders from across West and Central Africa. Given the high CCHFV seroprevalence, safeguards for human disease might include strengthening tick control interventions, surveillance in livestock markets, human serosurveillance among high-risk occupational groups (butchers, herders, veterinarians), and awareness programs for medical providers. The relatively lower RVFV prevalence does not preclude future outbreaks, especially in light of climate variability and increased transboundary trade.

## 5. Conclusions

This study provides a broad-scale evidence of concurrent exposure to CCHF and RVF viruses among ruminant livestock in Nigerian livestock markets. The findings highlighted a high CCHFV seropositivity, particularly in cattle and older female animals, and a lower but significant level of RVFV seropositivity, with geographic clustering in northern markets such as Illela. The clustering of seropositivity in high-contact nodes such as livestock markets emphasizes the risk of occupational exposure for herders, traders, butchers, and veterinarians.

These results suggested that both viruses are actively circulating in Nigeria, facilitated by ecological conditions favorable to vectors, livestock management practices, and cross-border animal movement. Strengthening integrated surveillance systems that link veterinary and public health sectors is therefore essential. Vector control, farmer awareness campaigns, and improved biosecurity at markets could mitigate transmission. Incorporating livestock markets into routine surveillance is particularly critical, as these nodes were shown to have elevated prevalence and represent high-risk contact points. The descriptive insights generated here highlight the need for analytical modeling to better understand independent and interacting risk factors that drive exposure in livestock populations.

## Figures and Tables

**Table 1:** Distribution of animals sampled by explanatory variables.

Variable	Category	Number	Percent
Species	Bovine	1150	33.3
	Caprine	1150	33.3
	Ovine	1150	33.3
Location	Bukuru livestock market	689	20
	Ganye livestock market	690	20
	Illela livestock market	690	20
	Maigatari livestock market	676	19.6
	Mubi livestock market	705	20.4
Age	Young	485	14.1
	Old	2963	85.9
Sex	Female	2208	64
	Male	1196	34.7
	Missing/Not recorded	46	1.3
Origin	Local	2410	69.9
	Foreign	1012	29.3
	Missing/Not recorded	28	0.8
Tick	No	2953	85.6
	Yes	449	13
	Missing/Not recorded	48	1.4

**Table 2:** Seroprevalence of CCHFV in ruminants across major livestock markets in Nigeria.

Variable	Total Samples	Total Positive (%)	95% CI
Overall seroprevalence	3450	936 (27.1)	25.6–28.6
Species			
Cattle	1150	637 (55.4)	52.5–58.1
Sheep	1150	200 (17.4)	15.3–19.7
Goats	1150	99 (8.6)	7.1–10.3
Sex			
Female	2202	670 (30.4)	28.5–32.4
Male	1188	257 (21.6)	19.4–24.1
Age			
Adult	2951	813 (27.6)	26.0–29.2
Young	482	123 (25.5)	21.8–29.6
Market location			
Illela	690	188 (27.2)	24.1–30.7
Maigatari	679	164 (24.3)	21.2–27.6
Mubi	705	250 (35.5)	32.2–39.1
Ganye	680	164 (24.1)	21.0–27.5
Bukuru	684	170 (24.9)	21.8–28.2
Origin of animals			
Local	1012	297 (29.3)	26.6–32.2
Foreign	2395	628 (26.2)	24.5–28.0
Presence of ticks			
Yes	449	180 (40.1)	35.6–44.7
No	2938	740 (25.2)	23.6–26.8

**Table 3:** Seroprevalence of RVFV in ruminants across major livestock markets in Nigeria.

Variable	Total Samples	Total Positive (%)	95% CI
Overall seroprevalence	3450	201 (5.8)	5.1–6.7
**Species**			
Cattle	1150	129 (11.2)	9.5–13.2
Sheep	1150	33 (2.9)	2.0–4.0
Goats	1150	39 (3.4)	2.5–4.6
Sex			
Female	2208	148 (6.7)	5.7–7.8
Male	1196	47 (3.9)	3.0–5.2
Age			
Adult	2963	171 (5.8)	5.0–6.7
Young	485	30 (6.2)	4.4–8.7
Market location			
Illela	690	77 (11.2)	9.0–13.7
Maigatari	676	50 (7.4)	5.6–9.6
Mubi	705	28 (4.0)	2.8–5.7
Ganye	690	35 (5.1)	3.7–7.0
Bukuru	689	11 (1.6)	0.9–2.9
Origin of animals			
Local	1012	107 (10.6)	8.8–12.6
Foreign	2410	91 (3.8)	3.1–4.6
Presence of ticks			
Yes	449	54 (12.0)	9.3–15.4
No	2953	144 (4.9)	4.2–5.7

**Table 4:** Mixed exposure of CCHFV and RVFV in ruminants across major livestock markets in Nigeria.

Variable	Total Samples	Total Positive (%)	95% CI
Cattle	1150	94 (8.2)	6.6–9.8
Sheep	1150	8 (0.7)	0.22–1.18
Goats	1150	2 (0.2)	0–0.46
Total	3450	104 (3.0)	2.4–3.6

## Data Availability

For reasons of confidentiality, the data used in this study are not published. Selected information may be shared with interested upon request and approval of the institute and herd owners.
